# Postoperative Magnetic Resonance Imaging (MRI) Scans for the Surgical Resection of Cranial Glial Tumors According to National Institute of Health and Care Excellence (NICE) Guidelines: A Single-Center Experience

**DOI:** 10.7759/cureus.51037

**Published:** 2023-12-24

**Authors:** Moudar Kouli, Arsalan Baig, Nicala Rampersad, Sonia Franchini, Preetam Upadhyaya, Sandra Balata, Babar Vaqas, Nikolaos Haliasos

**Affiliations:** 1 Neurosurgery, Queen's Hospital, London, GBR; 2 Orthopaedics and Trauma, Queen's Hospital, London, GBR; 3 Vascular Surgery, Queen's Hospital, London, GBR; 4 General Medicine, Damascus University, Damascus, SYR

**Keywords:** surgical resection, brain tumors, glioma, mri imaging, quality improvement project, nice guidelines, single-center, neurosurgery

## Abstract

Background

Glial tumours are the most common central nervous system (CNS) neoplastic lesions. They occur in 7 per 100,000 individuals in the United Kingdom (UK) and are categorized into astrocytomas, oligodendrogliomas, and glioblastomas in the adult population. The World Health Organization (WHO) has created a classification system in order to better categorise these lesions, placing them in a range from grade I to grade IV. The higher the grade, the poorer the prognosis. The National Institute of Health and Care Excellence (NICE) in the United Kingdom recommends that all surgical resections of glial brain tumours are followed by a postoperative magnetic resonance imaging (MRI) scan within a 72-hour to establish a baseline for further management.

Objective

We present a retrospective analysis that assessed the compliance rate with NICE guidelines among patients who underwent surgical resection of glial lesions at the Department of Neurosurgery, Queens Hospital Romford, between January 2022 and September 2023.

Materials and methods

A retrospective analysis was conducted on 136 glial tumour resections that were performed during the period between January 2022 and September 2023. The total time between the end of the operation and the MRI scan was calculated in hours for each procedure. This was analyzed into two groups with respect to compliance with the NICE guidelines, which are within 72 hours and after 72 hours. The non-compliant group was then further investigated regarding the reason for the delay. The cost related to delays was also determined by discussion with the hospital's finance department.

Results

All of the procedures were followed by a post-operative MRI scan but only 88% were within the timeframe recommended by NICE guidelines. The amount of delay was calculated in hours and the reasons for these delays were identified. We created two categories for delay: requesting delays and radiology department-related delays with an almost equivalent number of delays resulting from each category. This delay has resulted in approximately £19,845 of extra costs for inpatient stays.

Conclusion

A retrospective analysis at Queens Hospital, Romford, found good compliance with NICE guidelines for post-operative MRI scans in glial lesion resections from January 2022 to September 2023. Eighty-eight per cent of patients received scans within 72 hours, crucial for baseline assessment. A 12% non-compliance rate revealed areas for improvement, causing £19,845 in extra costs due to longer inpatient stays. Expediting scans to 36 hours could save around £30,876 annually and reduce complications like infections and thromboembolism. Proposed strategies include dedicated MRI slots and policy adjustments for MRI requests.

## Introduction

The National Institute of Health and Care Excellence (NICE) in the United Kingdom (UK) has issued guidelines for the optimal management of glial tumours [1]. According to these guidelines, all surgical resections of glial brain tumours should be followed by a postoperative magnetic resonance imaging (MRI) scan, which includes both pre- and post-contrast sequences within a 72-hour timeframe of the operation to establish a baseline for further management [1]. Gliomas are the most prevalent neoplastic lesion of the central nervous system (CNS) that occur in 7 per 100,000 individuals in the UK [2]. Gliomas are further categorized into astrocytomas, oligodendrogliomas, and glioblastomas [3]. The World Health Organization (WHO) has classified gliomas into four grades (I, II, III, IV), with Grade I indicating the most favourable prognosis; the prognosis worsens progressively as the grade gets higher [4]. NICE guidelines recommend surgical resection in specific cases to obtain histological diagnoses, which can aid in oncological treatment, and to resect as much of the tumour as is safely possible [1]. Moreover, the outcomes derived from the postoperative MRI scans can provide valuable insights into the potential for recurrence, advancement, and prognosis [5]. The most consequential data often emanates from MRI scans conducted within the timeframe of 24 to 72 hours following the operation [5,6]. A retrospective analysis assessed the compliance rate with NICE guidelines among patients who underwent surgical resection of glial lesions at Queens Hospital, Romford, Department of Neurosurgery, between January 2022 and September 2023. The adherence to guidelines significantly influences patient outcomes, as the importance of post-operative MRI is well-established in the literature, with studies indicating that accurate assessment of tumour resection extent is crucial for determining prognosis and guiding further treatment [7]. Evaluating compliance rates can highlight potential gaps in current practice, identify barriers to guideline adherence, and ultimately lead to enhanced patient care and outcomes. Given the complexity and variability of glial tumours, understanding how closely current practices at Queen's Hospital align with NICE guidelines provides essential insights into the quality of care and can drive improvements in clinical protocols and patient management strategies.

## Materials and methods

Retrospective data derived from a clinical audit done at Queens Hospital, Romford, was systematically collected relating to all patients who underwent surgical resection of gliomas during the period between January 2022 and September 2023. This specific timeframe was chosen to allow for an assessment of compliance with post-operative imaging guidelines, taking into consideration the potential influence of coronavirus disease 2019 (COVID-19) restrictions.

The department's operations archive underwent a thorough examination, filtering out surgeries unrelated to intracranial masses. Subsequently, the selected surgical cases underwent further review through operation notes, with a focus on exclusively including cases of surgical resections of intracranial tumours. The final list of cases was carefully selected by reviewing histology reports to ensure that only operations with histological findings of glial tumours were included, explicitly excluding cases limited to tissue biopsy.

The data collected included patient demographics, histopathology findings, surgical procedure dates and times, MRI scan dates and times, as well as the average number of admissions and the average cost of a patient's daily stay.

The data analysis process involved organizing the collected data into a Microsoft Excel spreadsheet (Microsoft Corporation, Redmond, WA, USA). Time intervals, measured in hours, between the conclusion of surgery and the subsequent MRI scan, as recorded in the hospital's system, were calculated. These time intervals were categorized into two groups: those within 72 hours and those beyond 72 hours, serving as a basis for assessing compliance with NICE guidelines [1]. Cases with MRI scans falling outside the recommended timeframe were closely examined to determine the likely causes of delay.

Additionally, the average time elapsed between surgery and the MRI scan was calculated using Excel's data processing capabilities. Delay causes were categorized into two groups: radiology departmental delay for scans requested prior to or within 24 hours post-operatively, and requesting delay for scans requested after 24 hours post-operatively. The request time was obtained from the hospital's radiology system, where this information is easily accessible. A retroanalysis of all MRI reports was conducted to identify the number of cases in which a residual was reported and those where it was not reported.

Financial information related to costs was obtained from the financial department at Queens Hospital, Romford.

## Results

Over a 21-month span, between January 2022 and September 2023, a total of 136 patients underwent surgical resection of glial tumours at Queens Hospital, Romford (Table 1).

**Table 1:** Expanded Results GBM: glioblastoma

	Sex	Age	Histology	Date of Surgery	Date of MRI	Hours	Within Target
1	Male	51	GBM	04/01/2022	06/01/2022	44	Yes
2	Male	58	GBM	06/01/2022	10/01/2022	93	No
3	Male	52	GBM	07/01/2022	09/01/2022	40	Yes
4	Male	48	Oligodendroglioma	11/01/2022	12/01/2022	25	Yes
5	Female	64	GBM	13/01/2022	15/01/2022	48	Yes
6	Female	53	GBM	14/01/2022	16/01/2022	47	Yes
7	Female	79	GBM	21/01/2022	23/01/2022	43	Yes
8	Male	49	GBM	21/01/2022	24/01/2022	69	Yes
9	Male	52	GBM	26/01/2022	28/01/2022	50	Yes
10	Female	46	GBM	31/01/2022	02/02/2022	49	Yes
11	Male	57	GBM	04/02/2022	06/02/2022	56	Yes
12	Female	66	GBM	4/02/2022	06/02/2022	42	Yes
13	Female	75	GBM	11/02/2022	14/02/2022	70	Yes
14	Male	58	GBM	11/02/2022	14/02/2022	68	Yes
15	Female	77	GBM	15/02/2022	17/02/2022	49	Yes
16	Female	72	GBM	25/02/2022	28/02/2022	72	Yes
17	Male	78	GBM	3/03/2022	07/03/2022	94	No
18	Male	58	GBM	03/03/2022	05/03/2022	43	Yes
19	Male	53	GBM	4/03/2022	06/03/2022	49	Yes
20	Female	62	GBM	4/03/2022	05/03/2022	20	Yes
21	Male	52	GBM	10/03/2022	11/03/2022	21	Yes
22	Male	62	GBM	15/03/2022	17/03/2022	49	Yes
23	Male	60	GBM	15/03/2022	17/03/2022	45	Yes
24	Male	67	GBM	18/03/2022	20/03/2022	48	Yes
25	Male	56	Oligodendroglioma	25/03/2022	27/03/2022	48	Yes
26	Female	74	GBM	28/03/2022	31/03/2022	68	Yes
27	Male	62	GBM	12/04/2022	14/04/2022	43	Yes
28	Male	61	GBM	15/04/2022	17/04/2022	41	Yes
29	Female	60	GBM	19/04/2022	22/04/2022	65	Yes
30	Female	58	GBM	22/04/2022	27/04/2022	115	No
31	Male	83	GBM	22/04/2022	24/04/2022	42	Yes
32	Male	61	Oligodendroglioma	29/04/2022	30/04/2022	17	Yes
33	Male	63	GBM	17/05/2022	19/05/2022	44	Yes
34	Male	65	Oligodendroglioma	24/05/2022	27/05/2022	70	Yes
35	Male	48	GBM	25/05/2022	26/05/2022	21	Yes
36	Male	36	GBM	27/05/2022	28/05/2022	20	Yes
37	Male	71	GBM	30/05/2022	01/06/2022	47	Yes
38	Male	76	GBM	31/05/2022	05/06/2022	140	No
39	Male	69	GBM	7/06/2022	09/06/2022	47	Yes
40	Male	41	GBM	17/06/2022	18/06/2022	21	Yes
41	Male	52	GBM	21/06/2022	23/06/2022	43	Yes
42	Male	77	GBM	24/06/2022	26/06/2022	42	Yes
43	Male	23	GBM	28/06/2022	29/06/2022	22	Yes
44	Male	64	GBM	4/07/2022	09/07/2022	118	No
45	Female	63	GBM	6/07/2022	10/07/2022	94	No
46	Male	57	GBM	22/07/2022	25/07/2022	70	Yes
47	Female	73	GBM	1/08/2022	05/08/2022	93	No
48	Male	23	GBM	2/08/2022	06/08/2022	92	No
49	Male	66	GBM	4/08/2022	06/08/2022	48	Yes
50	Male	54	GBM	10/08/2022	14/08/2022	96	No
51	Male	64	GBM	10/08/2022	12/08/2022	45	Yes
52	Male	56	GBM	12/08/2022	15/08/2022	68	Yes
53	Female	43	GBM	18/08/2022	20/08/2022	42	Yes
54	Female	79	GBM	1/09/2022	03/09/2022	44	Yes
55	Male	53	GBM	2/09/2022	03/09/2022	22	Yes
56	Male	63	GBM	7/09/2022	08/09/2022	23	Yes
57	Male	52	GBM	26/09/2022	28/09/2022	43	Yes
58	Female	67	GBM	29/09/2022	01/10/2022	49	Yes
59	Male	65	Astrocytoma	30/09/2022	04/10/2022	89	No
60	Male	57	GBM	30/09/2022	03/10/2022	69	Yes
61	Female	71	GBM	03/10/2022	05/10/2022	46	Yes
62	Male	67	GBM	07/10/2022	09/10/2022	45	Yes
63	Female	32	Astrocytoma	14/10/2022	15/10/2022	19	Yes
64	Male	66	GBM	25/10/2022	28/10/2022	72	Yes
65	Female	46	GBM	26/10/2022	30/10/2022	90	No
66	Female	59	GBM	28/10/2022	29/10/2022	17	Yes
67	Female	63	GBM	4/11/2022	07/11/2022	68	Yes
68	Male	41	GBM	11/11/2022	12/11/2022	22	Yes
69	Female	65	GBM	14/11/2022	17/11/2022	68	Yes
70	Male	74	GBM	15/11/2022	15/11/2022	1	Yes
71	Female	73	GBM	25/11/2022	27/11/2022	42	Yes
72	Female	66	Astrocytoma	29/11/2022	27/12/2022	670	No
73	Female	75	GBM	02/12/2022	05/12/2022	69	Yes
74	Male	62	GBM	08/12/2022	10/12/2022	46	Yes
75	Male	60	GBM	9/12/2022	11/12/2022	50	Yes
76	Male	80	GBM	12/12/2022	14/12/2022	40	Yes
77	Male	52	GBM	14/12/2022	15/12/2022	26	Yes
78	Male	73	GBM	15/12/2022	18/12/2022	66	Yes
79	Male	68	GBM	16/12/2022	18/12/2022	41	Yes
80	Female	64	GBM	29/12/2022	31/12/2022	48	Yes
81	Male	80	GBM	30/12/2022	31/12/2022	18	Yes
82	Male	26	Astrocytoma	6/01/2023	08/01/2023	42	Yes
83	Male	63	GBM	06/01/2023	07/01/2023	20	Yes
84	Female	61	GBM	11/01/2023	13/01/2023	45	Yes
85	Female	66	GBM	16/01/2023	17/01/2023	23	Yes
86	Male	52	GBM	21/01/2023	23/02/2023	42	Yes
87	Male	63	GBM	27/01/2023	29/01/2023	42	Yes
88	Male	64	GBM	29/01/2023	30/01/2023	26	Yes
89	Female	43	Astrocytoma	7/02/2023	08/02/2023	23	Yes
90	Male	58	GBM	10/02/2023	13/02/2023	71	Yes
91	Male	69	GBM	15/02/2023	17/02/2023	46	Yes
92	Male	55	GBM	16/02/2023	18/02/2023	49	Yes
93	Male	53	GBM	16/02/2023	18/02/2023	46	Yes
94	Female	64	GBM	21/02/2023	24/02/2023	68	Yes
95	Male	52	GBM	21/02/2023	23/02/2023	42	Yes
96	Female	66	GBM	23/02/2023	27/02/2023	95	No
97	Female	58	GBM	24/02/2023	27/02/2023	68	Yes
98	Male	73	GBM	03/03/2023	06/03/2023	71	Yes
99	Male	54	GBM	3/03/2023	05/03/2023	50	Yes
100	Female	77	GBM	6/03/2023	07/03/2023	20	Yes
101	Female	62	GBM	15/03/2023	17/03/2023	47	Yes
102	Male	48	GBM	17/03/2023	19/03/2023	41	Yes
103	Male	79	GBM	22/03/2023	24/03/2023	50	Yes
104	Female	73	GBM	24/03/2023	28/03/2023	99	No
105	Male	73	GBM	27/03/2023	30/03/2023	75	No
106	Female	65	GBM	20/04/2023	21/04/2023	24	Yes
107	Female	56	GBM	21/04/2023	25/04/2023	94	No
108	Male	56	Astrocytoma	21/04/2023	23/04/2023	42	Yes
109	Male	51	GBM	21/04/2023	22/04/2023	21	Yes
110	Female	55	GBM	21/04/2023	23/04/2023	44	Yes
111	Male	57	GBM	24/04/2023	25/04/2023	23	Yes
112	Male	70	GBM	28/04/2023	30/04/2023	52	Yes
113	Female	68	GBM	4/05/2023	05/05/2023	17	Yes
114	Male	62	GBM	10/05/2023	11/05/2023	18	Yes
115	Male	58	GBM	17/05/2023	19/05/2023	40	Yes
116	Male	57	GBM	19/05/2023	21/05/2023	49	Yes
117	Male	67	GBM	26/05/2023	28/05/2023	49	Yes
118	Female	63	GBM	30/05/2023	01/06/2023	45	Yes
119	Male	78	GBM	1/06/2023	03/06/2023	48	Yes
120	Male	67	GBM	2/06/2023	04/06/2023	47	Yes
121	Female	49	Astrocytoma	9/06/2023	11/06/2023	50	Yes
122	Female	66	GBM	15/06/2023	18/06/2023	66	Yes
123	Male	46	GBM	19/06/2023	21/06/2023	49	Yes
124	Female	69	GBM	24/06/2023	26/06/2023	48	Yes
125	Male	72	Astrocytoma	4/07/2023	07/07/2023	66	Yes
126	Male	73	GBM	10/07/2023	12/07/2023	45	Yes
127	Male	54	GBM	14/07/2023	16/07/2023	49	Yes
128	Female	71	GBM	24/07/2023	25/07/2023	23	Yes
129	Male	69	GBM	25/07/2023	27/07/2023	46	Yes
130	Male	25	Astrocytoma	1/08/2023	03/08/2023	45	Yes
131	Female	76	GBM	4/08/2023	06/08/2023	41	Yes
132	Male	46	GBM	8/08/2023	10/08/2023	42	Yes
133	Male	61	GBM	8/08/2023	10/08/2023	44	Yes
134	Male	49	GBM	18/08/2023	20/08/2023	43	Yes
135	Female	58	Oligodendroglioma	18/08/2023	21/08/2023	67	Yes
136	Male	68	GBM	22/08/2023	24/08/2023	44	Yes

**Figure 1:**
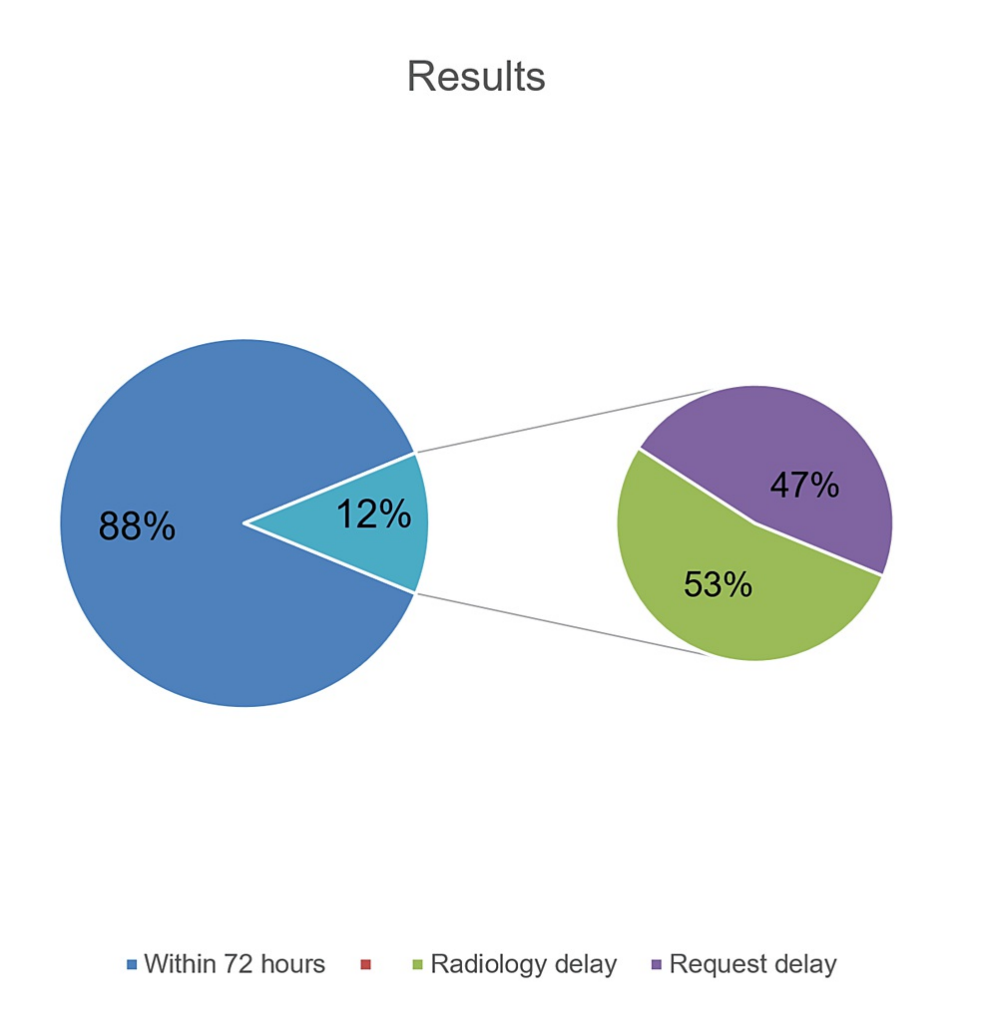
Results Summary The pie chart shows the results and the percentage of compliance and delays.

Among these patients, there were 89 (65%) males and 47 (35%) females with an average age of 61 years old ranging from 25-79 years old. Astrocytomas were diagnosed in 9 (7%) cases, with 5 falling under WHO Grade II and 4 classified as WHO Grade III [3]. Additionally, 5 (4%) patients were diagnosed with oligodendroglioma, with 3 categorized as WHO Grade II and the remaining 2 designated as Grade III [3]. The remaining 122 (89%) patients were histologically confirmed to have glioblastoma (GBM), classifying them as WHO Grade IV [3]. On average, patients had their post-operative MRI at 56 hours following their surgical procedures. Overall, 119 (88%) patients received their scan within the 72-hour window recommended by the NICE guidelines [1], and the remaining 17 (12%) patients did not meet the 72-hour timeframe with an average delay of 134 hours. Amongst the 17 patients who experienced delays, 9 (53%) were attributed to radiology departmental delay, while 8 (47%) resulted from delayed scan requests (Figure 1).

Of all 136 MRI scans that were reported, 48 (35%) showed no signs of residual tumour and the remaining 88 (65%) reported varying amounts of residual tumour. The average cost of a neurosurgery bed for 24 hours at our hospital is £470. The accumulative hours of delay were found to be 995 hours, which caused an extra cost of £19,845 for the inpatient stay.

## Discussion

Gliomas represent the most prevalent primary central nervous system tumours [2]. According to NICE guidelines [1], it is recommended that patients undergoing surgical resection of glial tumours should undergo post-operative MRI scans within a 72-hour timeframe [1]. This early post-operative imaging facilitates the establishment of a documented baseline, aiding in the subsequent planning of surgical and oncological interventions [1]. The postoperative MRI scan significantly contributes to predicting the likelihood of recurrence and overall survival, particularly when conducted within the recommended timeframe of 72 hours [5,6]. To assess compliance with these specific NICE guidelines [1], patient data from the Department of Neurosurgery at Queens Hospital, Romford, was systematically reviewed and analyzed. Instances of delays in post-operative scanning were investigated, with root causes categorized into two groups: radiology department delays and requesting delays. Overall, the analysis revealed a good rate of compliance (88%), subsequently leaving a 12% non-compliance rate. Of the previously mentioned 12%, an almost equivalent number of delays resulted from both requesting delays and radiology department-related delays. These delays could be substantially reduced with minor policy adjustments within both the radiology department and the surgical team.

A feasible option involves the allocation of a dedicated one-hour slot each day within the MRI department for neurosurgical imaging in which these scans can be performed. This allocation should not limit neurosciences imaging to this specific hour, and other scans should continue to be scheduled based on clinical prioritization, as is currently standard practice.

With regard to delays in requesting scans, a change to neurosurgical departmental policy could potentially eliminate any delay in submitting request forms. Assigning responsibility to a designated operating surgeon to attach an MRI request form to the printed operation note and place it in the patient’s notes can be an effective measure. A handover from the theatre team to the ward team can then be given that the imaging form needs to be submitted and where a completed form can be found. This would be particularly valuable in cases where a patient is transferred to the intensive therapy unit post-operatively and a breakdown of communication may occur between the neurosurgical junior doctors who may be based on the standard ward. A more substantial change would be the introduction of an electronic requesting system, allowing the operating surgeon to request an MRI concurrently with the creation of an operation note eliminating the delay entirely. However, this requires a significant change in culture and established processes at significant financial and organizational costs.

The neurosurgery department at Queens Hospital averages around 2.5 admissions per day, with the average cost of a neurosurgery bed for a 24-hour stay being £470. A total of 995 hours of delay in imaging were found in the period of study, resulting in approximately £19,845 of extra costs incurred. If the average time for a post-operative MRI scan was reduced to 36 hours from the current 55 hours, around £30,876 could be saved annually. This is also not accounting for the potential savings that could be wrought from non-glial tumours, which remains an area of potential further study.

In addition to the financial consideration, the health aspect is crucial. Extended hospital stay increases the risk of inpatient complications, such as infections and venous thromboembolisms, which might be potentially fatal. Performing an early MRI scan can shorten hospital stays participating in timely patient discharge which can help avoid these complications.

A further area of study that could be of interest and significance is to compare the current findings of this paper with the same methodology applied to a period of 21 months during the COVID-19 pandemic where there were greater restrictions and challenges in place to ensure compliance with national guidelines.

The early identification and quantification of residual tumours, when combined with histology findings, is invaluable for further management, both surgical and oncological management. A further retrospective study specifically quantifying the volume of pre-operative tumour and volume of potential residual post-resection with the correlation between prognosis and survival would be of benefit in highlighting the importance of early postoperative MRI.

Several limitations were faced while conducting this study. Firstly, it is important to acknowledge that this study was single-centred, which means that the findings might not be generalizable to broader settings. Secondly, the absence of prior research within the centre posed a challenge as there was no baseline to compare the results. Finally, the scarce existing literature on the subject limited the ability to compare the findings with other centres or countries.

## Conclusions

A retrospective analysis at Queens Hospital, Romford, found good compliance with NICE guidelines for post-operative MRI scans in glial lesion resections from January 2022 to September 2023. Eighty-eight per cent (88%) of patients received scans within 72 hours, crucial for baseline assessment. A 12% non-compliance rate revealed areas for improvement, causing £19,845 in extra costs due to longer inpatient stays. Expediting scans to 36 hours could save around £30,876 annually and reduce complications like infections and thromboembolism. Proposed strategies include dedicated MRI slots and policy adjustments for MRI requests can cause improvement in compliance.
